# Utility of a buccal swab point-of-care test for the *IFNL4* genotype in the era of direct acting antivirals for hepatitis C virus

**DOI:** 10.1371/journal.pone.0280551

**Published:** 2023-01-23

**Authors:** Aminata Sy, Leanne McCabe, Emma Hudson, Azim M. Ansari, Vincent Pedergnana, Shang-Kuan Lin, S. Santana, Marzia Fiorino, Aftab Ala, Ben Stone, M. Smith, Mark Nelson, Stephen T. Barclay, Stuart McPherson, Stephen D. Ryder, Jane Collier, Eleanor Barnes, Ann Sarah Walker, Sarah L. Pett, Graham Cooke, on behalf of the STOP-HCV-1 trial team

**Affiliations:** 1 MRC Clinical Trials Unit, University College London, London, United Kingdom; 2 Peter Medawar Building for Pathogen Research, Oxford, United Kingdom; 3 Mortimer Market Centre, Central and NorthWest London NHS Foundation Trust, London, United Kingdom; 4 Institute for Global Health, University College London, London, United Kingdom; 5 Clinical and Experimental Medicine, University of Surrey, Guilford, United Kingdom; 6 Infectious Diseases, Sheffield Teaching Hospitals NHS Foundation Trust, Sheffield, United Kingdom; 7 Hepatology, Brighton and Sussex Medical School, Brighton, United Kingdom; 8 HIV Medicine, Chelsea & Westminster NHS Trust, London, United Kingdom; 9 Gastroenterology, Glasgow Royal Infirmary, Glasgow, United Kingdom; 10 Hepatology, Newcastle Upon Tyne Hospitals NHS Trust, Newcastle upon Tyne, United Kingdom; 11 Hepatology, Nottingham University Hospitals NHS Trust, Nottingham, United Kingdom; 12 Translational Gastroenterology Unit, John Radcliffe Hospital, Oxford, United Kingdom; 13 Department of Infectious Disease, Imperial College London, London, United Kingdom; 14 NIHR Biomedical Research Centre, Imperial College NHS Trust, London, United Kingdom; Nigerian Institute of Medical Research, NIGERIA

## Abstract

**Background:**

The CC genotype of the *IFNL4* gene is known to be associated with increased Hepatitis C (HCV) cure rates with interferon-based therapy and may contribute to cure with direct acting antivirals. The Genedrive® *IFNL4* is a CE marked Point of Care (PoC) molecular diagnostic test, designed for in vitro diagnostic use to provide rapid, real-time detection of *IFNL4* genotype status for SNP rs12979860.

**Methods:**

120 Participants were consented to a substudy comparing *IFNL4* genotyping results from a buccal swab analysed on the Genedrive® platform with results generated using the Affymetix UK Biobank array considered to be the gold standard.

**Results:**

Buccal swabs were taken from 120 participants for PoC *IFNL4* testing and a whole blood sample for genetic sequencing. Whole blood genotyping vs. buccal swab PoC testing identified 40 (33%), 65 (54%), and 15 (13%) had CC, CT and TT *IFNL4* genotype respectively. The Buccal swab PoC identified 38 (32%) CC, 64 (53%) CT and 18 (15%) TT *IFNL4* genotype respectively. The sensitivity and specificity of the buccal swab test to detect CC vs non-CC was 90% (95% CI 76–97%) and 98% (95% CI 91–100%) respectively.

**Conclusions:**

The buccal swab test was better at correctly identifying non-CC genotypes than CC genotypes. The high specificity of the Genedrive® assay prevents CT/TT genotypes being mistaken for CC, and could avoid patients being identified as potentially ‘good responders’ to interferon-based therapy.

## Introduction

Human interferon lambda 4 (IFNL4) is a polymorphic pseudogene which, encodes the interferon (IFN) lambda 4 protein associated with human antiviral defences, particularly against hepatitis C (HCV). IFNL4 genotypes are strongly associated with spontaneous clearance of HCV and response to interferon treatment. Individuals with CC genotype of the single nucleotide polymorphism (SNP) ‘rs12979860 C/T’ have a two-fold higher response to interferon and ribavirin compared to TT genotype [1–3]. With the newer and highly efficacious direct acting antiviral (DAA) drugs, the impact of host genetics on treatment outcomes is not well characterized. However, relapse rates after shortened DAA therapy are significantly increased in individuals with the unfavourable TT genotype [4–6], suggesting a potential role for pre-treatment IFNL4 genotype testing in personalised treatment. The high cost of HCV treatment is a challenge for low resource settings. Point-of-care (PoC) technologies for genetic testing have the potential to inform individual treatment decisions may be of great importance especially in settings where access to sequencing facilities is limited.

The aim of this substudy was to compare the performance of both platforms with respect to IFNL4 status. The Genedrive® (previously Epistem) *IFNL4* genotyping platform is a small, handheld ‘Point of Care’ (PoC) molecular diagnostic device developed by Genedrive PLC UK. It is designed for in vitro diagnostic use to provide rapid, real-time detection of *IFNL4* SNP rs12979860 genotype status. The CE marked test [7] uses buccal swabs and provides a result within 60 minutes. The diagnostic kit is capable of analysing samples without the need of external information technology (IT) or specialised laboratory equipment by using real-time fluorogenic polymerase chain reaction (PCR) with end-point melt analysis. The CE mark was given after the robust performance against the conventional laboratory PCR genotyping test, with sensitivities and specificities of 100% and 100% respectively [8]. Advances in PoC technologies enable provision of rapid diagnostic results; real-time treatment decisions allowing for timely initiation of appropriate therapy and the potential to replace traditional lab-based assays. The PoC applications are global, though potential impacts may vary between resource-rich and resource-limited settings [9].

Although direct-acting antivirals (DAA) have largely replaced interferon/ribavirin treatment for hepatitis C virus (HCV), interferon could be of use with DAA therapy and in which a rapid *IFNL4* test may be clinically useful, especially as no blood draw is required. In addition, the standard 8–12 weeks DAA treatment courses, although short, may still represent over-treatment. Hence, several randomised controlled trials such as STOP HCV-1, conducted between 2017–2019 in the United Kingdom to cure mild chronic HCV genotype 1 or 4 infection [10] explored different approaches to DAA treatment shortening both in those with HCV-monoinfection and Human Immunodeficiency Virus (HIV)-HCV co-infection. *IFNL4* status seems to influence HCV cure with conventional durations of DAA, it has been reported, with 12 weeks treatment, patients with rs12979860 TT genotype were ∼4.5 times more likely to relapse than those with rs12979860 CC [11]. It is unknown whether *IFNL4* status is important in predicting HCV cure with shorter DAA courses. In addition, there are ongoing and trials in planning which are exploring short courses of interferon-based therapy in combination with DAA to only four weeks for selected genotypes [12]; therefore, the Genedrive® IFNL4 genotyping platform may still be relevant.

## Materials and methods

### Participant samples

STOP HCV-1, conducted between 2017–2019 in the United Kingdom, was a clinical trial assessing biomarker-stratified short-course first-line and re-treatment DAA oral regimens to cure mild chronic HCV genotype 1 or 4 infection. Participants could be HCV-monoinfected or HIV-HCV co-infected. HIV-co-infected participants were required to have HIV virological suppression (<50 copies/mL) and be on combination antiretroviral therapy. The study was conducted in accordance with the International Conference on Harmonization Good Clinical Practice Guideline and The Declaration of Helsinki, and the protocol approved by the East of England, Cambridge South Research Ethic Committee (15/EE/0435). All participants in the STOP HCV-1 trial were eligible for participation in this substudy of which, 120 provided written informed consent for genetic testing. Genetic testing included the buccal swab Genedrive® *IFNL4* genotyping assay and a blood draw for conventional host genetic sequencing (also with participant informed consent).

### Buccal swab genetic testing

The buccal swab testing, which is commercially not available was performed as per the kit instruction (7) by the Research Nurses at the clinical sites following consent. All research sites were provided with the Genedrive® *IFNL4* machine; nurses were supported, trained by the trial manager using training materials provided by Genedrive® and we were able to demonstrate feasibility across all sites. Following a mouth rinse with 30ml water, the buccal swab was rubbed across the participants inner cheek 10 times in a forward/backward motion. This was repeated using the reverse side of the swab on the opposite cheek. The swab tip was submerged in a clear container consisting of 1.8ml of lysis buffer provided with the assay kit, mixing for 30 seconds performed by the research nurses. 20μl of the buccal sample mix, which consisted of lysis buffer and the swab of inner checks of participants was pipetted into the neck of each tube of the Genedrive kit cartridge, the lid locked shut and the cartridge inserted into the Genedrive device for analysis. Results were obtained in 60 minutes, printed out and attached to the case report form. Each test was performed in triplicate within one sample cartridge. For a confirmed result to be achieved, at least two of the three tests had to return concordant results. If the results were discordant from the triplicate test, the site performed a repeat analysis.

### Host genotyping using the Affymetrix UK Biobank Axiom array

Whole blood collected in EDTA tubes was stored in 2ml Sarstedt cryovials at -80°C. Whole blood samples were subsequently shipped on dry ice to the central laboratory in Oxford for further sequencing. DNA was extracted from whole blood using the easyMAG automated nucleic acid extraction system by bioMérieux and quantified using Picogreen assay. DNA samples were processed using the Axiom 2.0 Assay Manual workflow on Axiom UK Biobank 96 well arrays. The Axiom UK Biobank array directly genotypes over 800,000 single nucleotide polymorphisms (SNPs) across the human genome, including rs12979860 (*IFNL4*)–probe ID AX-40445375. Data quality control was performed by the Oxford Genomics Centre using Axiom Analysis Suite software.

### Test accuracy for host genotyping using the Affymetrix UK Biobank array

The genotyping was performed using Affymetrix UK Biobank Axiom arrays, which includes SNP rs12979860. The raw intensity files were processed by Affymetrix Power Tool (version 2.10.2.2), following the standard guidelines provided by Thermo Fisher Scientific UK. Genotype data for SNP rs12979860 was extracted and the genotype calls and confidences for this SNP was examined. The genotype confidence for the samples was very high (posterior genotype probability of >0.97). Genotype calling was also confirmed independently by visual examination of the cluster plots by two researchers.

### Statistical analysis

The results (CC/CT/TT genotypes) of the buccal swab point-of-care test for the *IFNL4* rs12979860 SNP were compared with results generated from the Affymetrix UK Biobank array, and the sensitivity and specificity of the buccal swab test to detect CC vs non-CC genotypes were estimated. Binomial exact confidence intervals were estimated for all proportions. Analyses were performed using Statistical software for data science (Stata v15.1).

## Results

Buccal swab and whole blood samples for 120 participants were analysed. Baseline demographic, clinical, hepatitis C and where applicable, HIV, characteristics are detailed in (Table 1).

**Table 1:** Baseline demographic, clinical and viral characteristics.

Characteristics	N = 120
	Median (IQR) or n (%)
**Age** (years)	47 (39, 54)
**Sex**	
**Female**	36 (30%)
**Male**	84 (70%)
**Ethnicity**	
White	98 (82%)
South Asian	1 (1%)
Southeast Asian	2 (2%)
Hispanic/Latino	10 (8%)
Black Caribbean/American	2 (2%)
Black African	2 (2%)
Mixed	1 (1%)
Other	4 (3%)
**HCV genotype**	
1a	96 (80%)
1b	22 (18%)
4	2 (2%)
**Baseline HCV VL**	688137 (204763, 2112475)
**BMI (kg/m** ^**2**^ **)**	25 (22, 26)
**HIV co-infected**	47 (39%)
**-CD4+ T-cell count (cells/mm**^**3**^**)**	700 (540, 879)

Of the 120 participants, 84 (70%) were male, 98 (82%) were white with a median age of 47 years. The predominant HCV genotype was 1a (80%) and 47 (39%) were co-infected with HIV (Table 1). Participants were recruited from 11 different trial sites.

Whole blood genotyping identified 40 (33%) of participants with CC genotype, 65 (54%) CT and 15 (13%) TT. Buccal swab PoC testing identified 38 (32%), 64 (53%) and 18 (15%) respectively with CC, CT and TT *IL28B* genotype respectively.

Overall, results were similar between the two types of testing, with concordant results in 113 (94%; 95% CI 88%, 98%) participants (Table 2). Seven participants 6% (95% CI 2, 12) had discordant results. All participants with a TT genotype from whole blood genotyping were correctly identified as having a TT genotype on the buccal swab test, giving a sensitivity of 100% (97.5% CI 78%, 100%). For identifying the CC genotype compared to CT/TT using the buccal swab test (Table 3), sensitivity was 90% (95% CI 76%, 97%) and specificity was 98% (95% CI 91%, 100%). Both measures are high, though there was a trend towards the buccal swab test being better at detecting non-CC genotypes than CC genotypes (p = 0.08). There was no significant difference of having a concordant result between the three genotypes (chi-squared p = 0.31).

**Table 2:** Comparisons between Genedrive buccal swab and whole blood genotyping: CC, CT, and TT.

Genedrive® result	Result from whole blood genotyping			Total
	CC	CT	TT	
CC	36	2	0	38 (32%)
CT	2	62	0	64 (53%)
TT	2	1	15	18 (15%)
**Total**	40 (33%)	65 (54%)	15 (13%)	120

**Table 3:** Comparisons between Genedrive buccal swab and genome-wide genotyping: CC vs. non-CC.

Genedrive® result	Genome sequencing result		Total
	CC	CT/TT	
CC	36	2	38
CT/TT	4	78	82
Total	40	80	120

## Discussion

In this substudy of STOP-HCV-1, *IFNL4* genotypes using the CE marked Genedrive® (buccal swab test) were identified with a 94% accuracy compared to whole blood genotyping using the Affymetrix UK Biobank array, with sensitivity and specificity similarly high. Overall, we detected a slightly lower sensitivity than that reported by Duffy et al [13] who used the Taqman platform. It is unclear what the drivers of these discordant results are.

The study represents a substudy of the overall STOP-HCV-1 trial, as such we cannot be sure that this may introduce bias in performance of the buccal swab test results. This is because the sub sample may not have a representative set of genotypes compared to the overall study population.

However, the genotypes distribution in this substudy does not differ greatly from the main trial, where 32%, 55% and 13% of participants had genotyping results of CC, CT and TT respectively. The seven discordant results out of 120 were from samples taken at several different clinics and are therefore unlikely to be the result of user error. Genotypes data generation using arrays are based on clustering genotyping results from several samples (best practices recommend more than 90 samples). When genotyping is successful, the genotype calls, i.e., the proportion of samples for which a genotype is called, should be above 95% and the confidence scores below 0.15. Genotypes called achieving these criteria can be considered to be gold standard. The seven discordant genotypes in our study achieved these criteria. Although we cannot exclude that discordance was due to a lower quality of DNA material for these seven samples (not detected by our standard checks), it is more likely that the CE marked Genedrive® failed. The ability to perform a discrepancy analysis was limited by two factors, the analysis was performed sometime after swab testing took place, and the sequencing was all done at the end of the trial.

In our study, sensitivity was lower than specificity, but still fairly high at 90%, indicating that the buccal swab test was better at determining non-CC genotypes compared to CC genotypes, although this was not statistically significant. The high specificity of the Genedrive® assay prevents CT/TT genotypes being mistaken for CC, and therefore prevents patients being potentially undertreated with an increased risk of treatment failure.

The lower sensitivity would potentially lead to misclassification of CT/TT resulting in overtreatment in 10% of patients, which could place unnecessary burden on patients and resources, but is less of a concern than undertreatment, which would occur in 3% of patients. In a setting where interferon-based therapy is used, misclassification of CT/TT could have clinical care implications because of overtreatment resulting in increased toxicities. However, patient outcome in terms of virology should not be compromised.

Although *IFNL4* polymorphisms are important predicators of hepatitis C cure with interferon-based therapies, newer DAA therapies are interferon-free, and as such *IFNL4* polymorphism assessment will not be as useful in countries where only DAAs are being used to treat HCV [14,15]. However, there are still countries around the world with limited or no access to DAA, where interferon-based treatments are still being used [16]. In this setting, a PoC buccal test could be very useful in discerning whether the patient has favourable or unfavourable *IFNL4* genotype with respect to interferon responsiveness. The clinical utility of *IFNL4* genotype with respect to achieving HCV cure with shortened DAA treatments remains under evaluation [17].

The advantages of the Genedrive® PoC assay compared to the host genotyping is that it is easy to use, requires no trained specialists and is low cost. Moreover, it is non-invasive as it uses buccal swab samples instead of blood, and results are readily available in less than hour making it highly suitable for same day clinical decisions.

In summary, the Genedrive® PoC *IFNL4* assay performed well against standard blood-based host genotyping which if gold standard. The test may still be relevant as a clinical tool where interferon-based treatments are still being utilised in combination with DAAs for the treatment of Hepatitis C, and further data is being gathered on its role in predicting cure with shortened DAA treatment regimens with and without interferon. Advances in PoC technologies looks promising and has clinical implication for which genotype- based diagnostics is becoming increasingly useful.
